# IMPACT OF DUAL-TASKING AND DEMENTIA DISEASE SEVERITY ON POSTURE, GAIT, AND FUNCTIONAL MOBILITY IN RESIDENTIAL CARE

**DOI:** 10.1093/geroni/igad104.3412

**Published:** 2023-12-21

**Authors:** Deborah Jehu, Ryan Langston, Richard Sams, Lufei Young, Mark Hamrick, Haidong Zhu, Yanbin Dong

**Affiliations:** Augusta University, Augusta, Georgia, United States; Augusta University, Augusta, Georgia, United States; Augusta University, Augusta, Georgia, United States; University of North Carolina at Charlotte, Charlotte, North Carolina, United States; Medical College of Georgia, Augusta, Georgia, United States; Medical College of Georgia, Augusta, Georgia, United States; Medical College of Georgia, Augusta, Georgia, United States

## Abstract

Dual-tasking may be a useful tool for fall-risk screening among people living with dementia (PWD), but more evidence is needed. Our aims were to compare single- and dual-task posture, gait and functional mobility; and examine the relationship between dementia severity and dual-task interference among PWD. Thirty PWD (Age:81.8±7.3years; 35% Female; Montreal Cognitive Assessment:10.5±6.1points) from two residential care facilities wore six APDM inertial sensors and completed two trials of single- (feet apart) and dual-task posture (feet apart while counting backwards by 1’s), single- (walk 4m) and dual-task gait (walk 4m while naming words), and single- (timed-up-and-go (TUG)); and dual-task functional mobility (TUG while completing a category task). Results revealed that greater frequency, jerk, and area were observed during dual- than single-task posture (ps< 0.05), with no differences in velocity. Slower gait speed, greater double limb support, shorter stride length, and reduced elevation at mid-swing were detected during dual- relative to single-task gait (ps< 0.05), with no differences in medial-lateral trunk sway. Dual-tasking resulted in a longer duration, reduced turn angle, and slower turn velocity than single-tasking for the TUG (ps< 0.05), with no differences in sit-to-stand lean angle. Dual-task interference (greater jerk, faster gait speed) was related to moderate-to-severe compared to mild dementia (ps< 0.05). Dual-tasking was more sensitive to detect impairments in posture, gait, and functional mobility than single-tasking. Moderate to severe PWD had poorer dynamic stability and a reduced ability to appropriately select cautious gait during dual-tasking than those with mild dementia, which should be considered in fall-risk screening.

